# Immersive Simulation Training in Health Informatics: Qualitative Descriptive Study

**DOI:** 10.2196/106961

**Published:** 2026-09-17

**Authors:** Jennifer Skelton, Savannah Siebers, Alanna Pelmore

**Affiliations:** 1 Department of Health Informatics and Information Management, University of Southern Indiana, 8600 University Blvd, Evansville, IN, 47712, United States, 1 812 465 1036

**Keywords:** simulation training, virtual reality, qualitative research, change management, end user training, health informatics

## Abstract

This qualitative research study offers preliminary evidence that immersive simulation training can influence change management skills, confidence, and perceived usefulness among health informatics professionals.

## Introduction

As health care technology success relies on people as much as design, preparing health informaticists to navigate stakeholder dynamics and sustain change is critical. However, when it comes to transformation in health care, the industry has an “implementation problem” because achieving success, meaning that desired outcomes are realized, has become difficult [1]. A systematic review of the change management practices and outcomes literature found that the choice of management practice and leaders’ engagement with followers were important differentiators between project success and failure [1]. Commonly used models include Kotter’s 8-Step Process, the Prosci Methodology, and the PEPE model [2-4].

Employers of health informaticists recognize this need for “soft skills” for health IT implementation. Flite et al [5] analyzed job advertisements for health care data analysts, a subset role within health informatics. The researchers found that 62% of postings desired applicants with interpersonal skills, and 6% specifically called for change management skills [5].

The development of virtual reality (VR) technology has enabled clinical skill development in health care education and practices [6]. Experiences with authentic simulations are considered a motivating factor in VR health care scenarios, and VR can also provide a safe environment for professionals to practice essential nonclinical skills [6]. Although simulation is used effectively in health care education, research on developing change management skills among health informaticists remains underexplored.

This study aimed to generate preliminary data on health informaticists’ perceptions of how immersive simulation training influences change management skills, confidence, and decision-making.

## Methods

### Study Design

**Figure 1.**
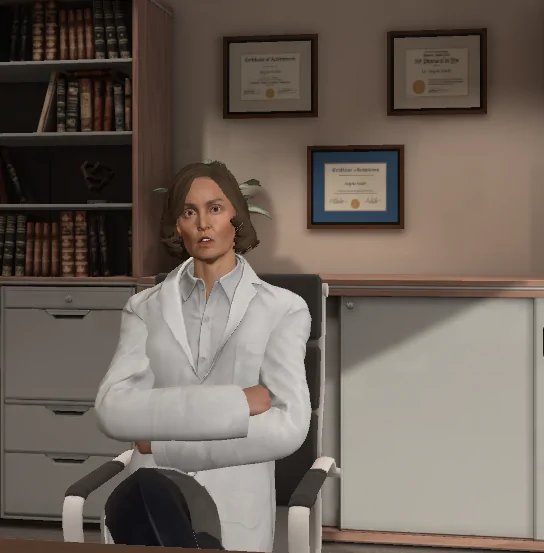
Cornerstone Immerse interaction with Wanda, MD.

This qualitative descriptive study explored participants’ experiences and confidence in skill development after completing an immersive simulation. Data were collected from February 13 to March 20, 2026. Of 14 people purposively recruited via snowball sampling, 9 health informatics professionals provided informed consent and completed either a desktop web-based or head-mounted display (Meta Quest 3) version of the simulation, custom-built using the Cornerstone Immerse CoPilot Designer, which enables emotionally realistic role-play with virtual human characters [7] (Figure 1).

After simulation completion, participants concluded with a semistructured online Qualtrics interview. The three coauthors conducted reflexive thematic analysis by each familiarizing themselves with the data, iteratively summarizing responses into categories at weekly meetings, developing and reviewing themes, and contextualizing findings against the study aims, with disagreements resolved by consensus [8]. Weekly discussions focused particularly on responses to questions addressing confidence, decision-making, application to daily work, and usefulness, allowing the team to track the depth and consistency of responses across interviews. The team collectively decided to discontinue data collection after 9 participants, judging that continued interviews were enriching existing categories rather than substantively expanding them.

The change management module “Guiding Lasting Change” was created to allow real-time conversations with change-resistant health care staff in a realistic health IT change scenario. Informed by various change management models [2-4], the simulation provided structured practice in core interpersonal skills essential for health informatics professionals, including communication, conflict management, self-awareness, and understanding others’ perspectives.

The module used instructional scaffolding during gameplay. Participants began by observing Jade, the nurse manager of a cardiac unit, delivering a discharge-planning project update. After a brief change management knowledge lesson from two virtual coaches, participants role-played as health informatics consultants negotiating one-on-one interactive exercises with Wanda, MD, and Keegan, PharmD. These change-resistant clinical team members allowed participants to practice active listening, empathy, and asking probing questions. Participants began the interactions by selecting from branching, dialogue-driven exchanges accompanied by immediate feedback, then advanced to an open-ended, unscripted dialogue with a role-based prompted AI avatar. A final postassessment activity with James, RN, provided participants with scored feedback. The entire 5-lesson module was completed in an average of 12.3 minutes.

### Ethical Considerations

Ethics approval was obtained from the institutional review board of the University of Southern Indiana, Evansville, Indiana (2411349‐1). Written informed consent was obtained from all participants before enrollment. Participant confidentiality was maintained by using deidentified data.

## Results

Table 1 shows participant insights from the simulation intervention. Themes around confidence, decision-making, daily practice, and usefulness emerged. Health informaticists reported increased confidence in change management skills after the simulation. Approximately one-third of participants said that open-dialogue practice with the pharmacist AI avatar influenced their change management skills due to the critical thinking required and the way it reflected real-time conversations. Participants said the simulation influenced decision-making in change-related situations by reinforcing active listening, consideration of end user perspectives, and empathy. Participants described the experience as more engaging and realistic than traditional training methods, such as videos or readings, and said it prepared them for difficult conversations with end users of implementation projects. Several participants noted that verbal, free-response interactions were particularly effective at simulating real-world scenarios.

**Table 1.** Study themes and supporting participant quotes.

Theme	Participant quote
Confidence	“I feel more confident in my ability to manage the social aspects of change management... I’m used to the logistical concerns, but I tend to struggle with the social side.”
Usefulness	“Simulating a scenario where you practice working with frustrated end users makes you more prepared when you have to address this in real life... the verbal option more closely simulates real life and requires you to think on your feet.”
Decision-making	“It gave real-time interactions that forced me to think quickly... it emphasized empathy, active listening, and reinforced the need for open-ended questions.”
Daily practice	“I will continue to use what I was taught when it comes to listening, asking questions, and showing empathy... I want to make sure I handle every situation gracefully.”

## Discussion

### Principal Findings

Overall, these results suggest that immersive simulation training could be an effective skill-building tool for developing change management competencies and may serve as a valuable addition to professional development in health informatics. The immersive simulation enabled practiced engagement with followers, laying a foundation for project success. Participants expressed that the simulation allowed them to apply empathy and critical thinking to mitigate end user frustration and coformulate solutions, and they emphasized that the simulation helped them recognize the importance of de-escalation practices in handling change-related situations productively.

Participants reported intending to apply active listening strategies in daily practice to address concerns and problem-solve with resistant coworkers—interpersonal skills identified as desirable in health informatics job postings [5]. They also described the authentic hospital simulation as useful for entry-level professionals learning to acknowledge end user frustration, further supporting VR’s value as a safe practice environment [6]. These findings support future research into lower-cost immersive simulation and AI alternatives to help health informatics professionals build the change management skills needed for sustained health IT project success.

Although snowball sampling ensured reach among active health informatics professionals, nonprobability sampling may introduce sampling bias, limiting generalizability and the diversity of viewpoints reported [9]. Findings reflected immediate, self-reported perceptions and do not demonstrate long-term behavior change or skill acquisition.

### Conclusions

This study has laid the groundwork for future research while highlighting the potential value of developing immersive simulation training modules for nonclinical, change management, and interpersonal skill development among health informatics professionals.

## Abbreviations

VR: virtual reality
